# Epidemiological Characterisation of *bla*_NDM_-Positive *Enterobacterales* from Food-Producing Animal Farms in Southwest China

**DOI:** 10.3390/microorganisms11092304

**Published:** 2023-09-13

**Authors:** Renqiao Wen, Hongcheng Wei, Tiejun Zhang, Peng Ma, Qin Wang, Chao Li, Zhonghan Li, Changwei Lei, Hongning Wang

**Affiliations:** 1 Laboratory of Bio-Resource and Eco-Environment, Ministry of Education, College of Life Sciences, Sichuan University, Chengdu 610017, China; 2 Animal Disease Prevention and Food Safety Key Laboratory of Sichuan Province, Chengdu 610064, China

**Keywords:** antibiotic resistance, *Enterobacterales*, Carbapenemase, *bla*_NDM_, transmission

## Abstract

Carbapenems are atypical β-lactam antibiotics with a broade antibacterial spectrum and strong antibacterial activity; however, the emergence and spread of carbapenemases have led to a decline in their effectiveness. New Delhi metallo-β-lactamase (NDM) is an important carbapenemase that has attracted widespread attention and poses a major threat to public health. To investigate the epidemiological characteristics of *bla*_NDM_ in swine and chicken farms in southwestern China, we isolated 102 *bla*_NDM_-positive *Enterobacterales* strains from 18 farms in Sichuan and Yunnan provinces in 2021, with *Escherichia coli* and *Klebsiella* spp. being the main reservoirs of *bla*_NDM_, variant *bla*_NDM_-5 being the most prevalent, and all strains being multi-drug resistant. Whole-genome sequencing analysis of 102 *bla*_NDM_-positive *Enterobacterales* strains revealed that *bla*_NDM_ had spread primarily through its carriers on the same farm and among the 18 farms in this study. A high degree of genetic similarity between animal-derived *bla*_NDM_-positive *Escherichia coli* strains and human-derived strains was also identified, suggesting a potential mutual transmission between them. Nanopore sequencing results indicated that *bla*_NDM_ is predominantly present on the IncX3 plasmid, that an insertion sequence might be important for recombination in the *bla*_NDM_ genetic environment, and that most of the plasmids carrying *bla*_NDM_ are transferable. Collectively, our results enrich the current epidemiological information regarding *bla*_NDM_ in pig and chicken farms in Southwest China, revealing its transmission pattern, as well as the potential risk of transmission to humans, which could help to better understand and control the spread of *bla*_NDM_.

## 1. Introduction

The issue of antibiotic multi-resistance in bacteria is crucial because of the significant threat it poses to human health [1]. Carbapenems are considered important antibiotics for use against multi-drug-resistant (MDR) bacteria (resistant to at least three types of antibiotics). However, after carbapenems’s clinical use, associated resistant strains have started to become prevalent [2,3]. Three classes of carbapenemases play a major role in the prevalence of carbapenem-resistant bacteria, namely class A (KPC, SME, GES, etc.), class B (IMP-, VIM-, and NDM-like metallo-β-lactamases (MβLs)), and class D (OXA) [4]. New Delhi metallo-β-lactamase (NDM) is an MβL that hydrolyses most β-lactams, but not monobactams [5]. *bla*_NDM_-_1_-carrying strains were identified in hospital patients in India in 2009, and since then, strains carrying this gene or its variants have been widely identified in other countries [6,7]. Moreover, in recent studies, NDM appeared to be a significant cause of bacterial resistance to carbapenems [5,8,9,10,11].

Although the use of carbapenems has never been allowed in the animal breeding process, the current percentage of livestock farm animals carrying NDM-positive strains cannot be ignored [12]. However, because of the close relationship between farmed animals and humans, these *bla*_NDM_-positive strains can still be transmitted to humans, representing a potential threat to human health. Therefore, monitoring animal-derived *bla*_NDM_-positive strains is of public health importance.

In this study, to refine the investigations of animals carrying NDM-positive bacteria in pig and chicken farms in the Sichuan and Yunnan provinces of China and to assess the transmission pattern of the *bla*_NDM_ gene among them and to assess the risk of transmission to humans via farmed animals, we collected samples from these locations. Here, 102 NDM-positive strains were isolated from 1512 samples, and we evaluated their prevalence and molecular epidemiological characteristics. We also assessed the transmission pattern of the *bla*_NDM_ gene by combining the relevant information in the database.

## 2. Materials and Methods

The animal experiment operations involved in this study were maintained in compliance with the Approval from the Medical Ethics Committee of Sichuan University (License: K2022018).

### 2.1. Sample Collection, Strain Isolation, and Identification

The samples in this study (*n* = 1512) were obtained from pig (*n* = 942) and chicken farms (*n* = 570) in the Sichuan and Yunnan provinces, and the types included fecal and anal swabs (Supplementary Table S1). The samples were transported in a cryopreserved state, and anal swabs and 0.5 g stool samples were taken and placed in 10 mL of BHI broth medium (Luqiao, Beijing, China) at 37 °C, with shaking at 180 rpm, and incubated for 12 h. Then, 100 µL of medium was spread onto EMB (Eosin Methylene Blue, Luqiao, Beijing, China) agar medium containing 1 μg/mL meropenem and cultured at 37 °C for 16 h, and 1~2 colonies of different shapes were picked for purification and culture. The 16S rRNA and *bla*_NDM_ genes of the purified strain were amplified using PCR and sequenced [13,14].

### 2.2. Antibiotic Susceptibility Testing

The broth microdilution method recommended by the Clinical and Laboratory Standards Institute (CLSI) was used to determine the susceptibility of all strains to the following antibiotics: gentamicin (GEN), florfenicol (FFC), polymyxin B (PMB), ciprofloxacin (CIP), cefoxitin (FOX), fosfomycin (FOS), aztreonam (ATM), doxycycline (DOX), norfloxacin (NOR), tetracycline (TET), tigecycline (TGC), ceftazidime (CAZ), amoxicillin/clavulanate (2:1) (AMC), ceftriaxone (CRO), amikacin (AMK), trimethoprim-sulphamethoxazole 1:19 (SXT), and meropenem (MEM). Interpretation was conducted according to the relevant resistance criteria in the CLSI document M100-S22 [15,16]. *Escherichia coli* ATCC25922 was used as the quality control strain.

### 2.3. Whole Genome Sequencing and Assembly

Genomic DNA of 102 NDM-positive strains was extracted using the Tiangen Bacterial DNA Extraction Kit. The entire genomic DNA was fully sequenced using the Illumina HiSeq system (Novogene, Tianjin, China), and raw data were evaluated for quality and filtered by fastaQC v0.12.1 and Cutadapt v4.2 [17,18]. Clean reads were assembled into contigs using SAPdes v.3.15.2 [19]. Whole genome sequencing of strains was performed using the Oxford Nanopore GridION (Novogene, Tianjin, China) and Illumina platforms. Using unicycler v0.5.0 [20], Illumina platform sequencing data and Nanopore sequencing data were subjected to mixed stitching.

### 2.4. Bioinformatics Analysis

The nucleotide sequence files of strains classified according to different species were annotated using Prokka v1.14.5 [21], and the annotated files were used as the input for roary v3.13.0 [22] to obtain the core genome comparison sequences. The phylogenetic tree was further reconstructed randomly based on the maximum likelihood method using iqtree v2.1.2 [23] with the default parameters. MLST v2.23.0 (https://github.com/tseemann/mlst, 19 November 2022) was used to analyze the ST types of all of the strains [24]. The antibiotic resistance gene-related information of the isolates was predicted using ResFinder 4.0 [25]. The plasmid replicon species were determined via an analysis using PlasmidFinder 2.1 [26]. The minimum spanning tree of the multilocus sequence typing (MLST)-based isolates was constructed using GrapeTree [27]. GView Server (https://server.gview.ca, 9 December 2022) was used to generate pan-plasmid sequences of similar plasmids [28]. Visualization and embellishment of the phylogenetic tree were performed using ChiPlot [29]. Circle maps of the plasmids were generated using Proksee [30]. Gene cluster comparison maps were obtained using Clinker v0.0.27 [31].

### 2.5. Conjugation Experiment

The filter mating method was used to perform the conjugation experiment, and *E. coli* J53, which is resistant to sodium azide, was used as the recipient to assess the *bla*_NDM_ gene transfer capacity from the *bla*_NDM_-positive strain to the recipient. The donor and recipient cells were cultivated in Luria–Bertani medium (Luqiao, Beijing, China) until they reached the logarithmic growth phase. They were then mixed at a 1:3 ratio and spread on a sterile 0.22-micron filter on a Luria–Bertani agar plate, where they were incubated for 24 h at 37 °C. Transformants were selected on LB agar plates with 4 mg/L meropenem and 100 mg/L sodium azide, and PCR-based confirmation was performed. The number of transformants divided by the total number of recipient bacteria was used to calculate the transfer frequency.

### 2.6. Data Availability

Information on the strains containing *bla*_NDM_-1 and *bla*_NDM_-5 variants (Dates to December 2022, Country: China, Species: *E. coli*, *K. pneumoniae*, *K. quasipneumoniae*, *P. vermicola*) obtained from the GenBank database is detailed in Supplementary Table S2. The nucleotide sequences of the bacterial genomes obtained from this study collection were deposited into the NCBI database and are publicly available under the accession number PRJNA909031. The NCBI database accession numbers of the plasmids carrying the *bla*_NDM_ gene were OQ230782–OQ230789, OR233054-OR233062.

## 3. Results

### 3.1. Prevalence of bla_NDM_-Positive Bacteria in Farm Animals

Here, 102 *bla*_NDM_-positive strains (57 pigs and 45 chickens) were isolated from 1512 fecal or anal swab samples (942 pigs and 570 chickens) from 18 farms in the Sichuan and Yunnan provinces in 2021 (Detailed information in Supplementary Table S1). The total incidence of *bla*_NDM_-positive strains in animals was 6.75%, comprising 6.05% in pigs and 7.89% in chickens, which suggests that the *bla*_NDM_ gene is carried at a high rate in farm animals. The 102 strains included 57 strains of *E. coli*, 31 strains of *Klebsiella quasipneumoniae*, 9 strains of *Klebsiella pneumoniae*, 4 strains of *Providencia vermicola*, and 1 strain of *Proteus mirabilis*. Two *bla*_NDM_ subtypes were identified through further sequencing, namely *bla*_NDM_-1 (29, 28.5%) and *bla*_NDM_-5 (73, 71.6%). *bla*_NDM_-5 was determined to be the main common variant. Details of this are presented in Supplementary Table S1.

### 3.2. Antimicrobial Susceptibility, Antibiotic Resistance Genes, and Plasmids of NDM-Positive Strains

**Figure 1:**
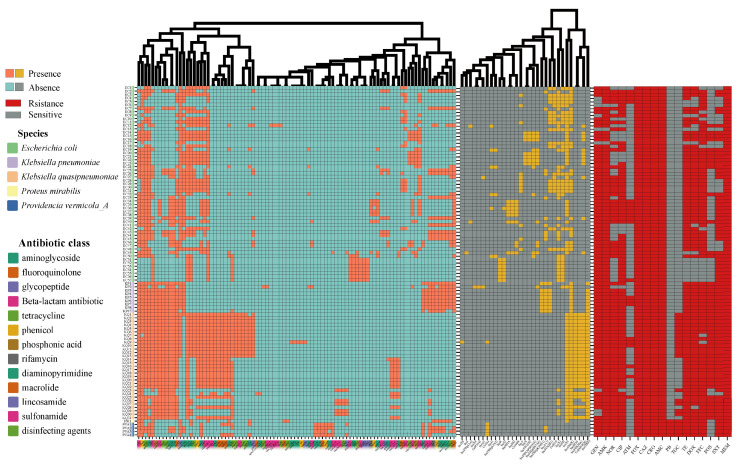
Heat map of resistance genes, plasmid replicons, and antibiotic resistance phenotypes of 102 *bla*_NDM_-positive strains. Orange and yellow: present; blue and grey: absent; red: resistant; grey: sensitive. Clustering of resistance genes and plasmid replicons on the *x* axis.

Phenotypic results of the 102 NDM-positive strains based on resistance to 17 antibiotics showed that these strains were MDR (Figure 1, Supplementary Table S3). These strains showed very high rates of resistance to GEN, AMK, CIP, TET, FFC, SXT, and NOR, reaching 91.2%, 97.1%, 86.3%, 85.3%, 86.3%, and 71.6%, respectively. All of the strains were resistant to FOX, CAZ, CRO, MEM, and AMC. Some isolates had MICs up to 64 µg/mL for meropenem. *K. pneumoniae* was completely resistant to GEN, AMK, TET, FFC, FOS, and SXT. *K. quasipneumoniae* was also resistant to AMK, NOR, CIP, and TET at a rate of approximately 100%. Of note, the rate of TGC resistance for *K. quasipneumoniae* strains in this study reached 58.1% (18/31), which is high. *E. coli*, *K. pneumoniae*, and *K. quasipneumoniae* were all found to be susceptible to PMB.

According to the genome analysis results, 94 drug-resistant genes in 13 categories were found in the *bla*_NDM_-positive strains (Figure 1, Supplementary Table S4). We found that different bacterial species displayed different antibiotic-resistance genotypes. The antibiotic-resistance genes with a high prevalence in *E. coli* were *aac(3)-Iid* (63.2%), *aadA2* (68.4%), *aph(3’)-Ia* (73.7%), *tet*(A) (77.2%), *floR* (78.9%), *ble* (84.2%), *dfrA12* (68.4%), and *sul3* (61.4%). Meanwhile, *sul1*, *sul2*, *floR*, *tet*(A), *aph(3′)-Ia*, *fosA*, *blaLAP-2*, *oqxB*, *oqxA*, *aph(6)-Id*, *aph(3″)-Ib*, *qnrS1*, *ble*, *aac(3)-Iid*, *dfrA1*, *bla*_NDM-1_, *arr-3*, *catB3*, *dfrA27*, *aadA16*, *aac(6’)-Ib-cr5*, *qacEdelta1*, and *mph(A)* were present in almost all *K. pneumoniae* genomes (>88.9%). Furthermore, *sul1*, *sul2*, *floR*, *tet*(A), *fosA*, *blaLAP-2*, *oqxA*, *oqxB*, *aph(6)-Id*, and *aph(3″)-Ib* resistance genes were present in almost all (>95%) *K. quasipneumoniae* isolates. Finally, the antibiotic resistance genes *tet*(X4) (16.1%, five strains from four farms) and tmexC1-tmexD1-toprJ1 (41.9%, 13 strains from 7 farms), related to tigecycline, an important clinical antibiotic, were highly prevalent in *K. quasipneumoniae* (Supplementary Table S4).

Furthermore, plasmid sequence analysis showed that the 102 *bla*_NDM_-positive strains carried 27 types of plasmids (Figure 1, Supplementary Table S4). Five plasmid types, IncFIB (66.7%), ColE10 (35.1%), IncFII (52.6%), IncX1 (61.4%), and IncX3 (66.7%) in *E. coli* were present at higher rates. Almost all *K. pneumoniae* isolates carried IncA/C2 (88.7%), IncFIA (HI1) (88.7%), IncFIB (100%), and IncFII (K) (88.7%) types. Moreover, IncFIB, IncFII(K), and IncX3 were detected in all *K. quasipneumoniae* isolates. Col (MG828) (87.1%) and IncHI1B (83.9%) also exhibited a high prevalence among the *K. quasipneumoniae* strain. Overall, IncFIB (76.5%), IncX3 (69.6%), Col (MG828) (38.2%), IncFII(K) (38.2%), and IncX1 (39.2%) were the most commonly carried plasmid types in *bla*_NDM_-positive strains in this study.

### 3.3. MLST and Phylogenetic Analysis

**Figure 2:**
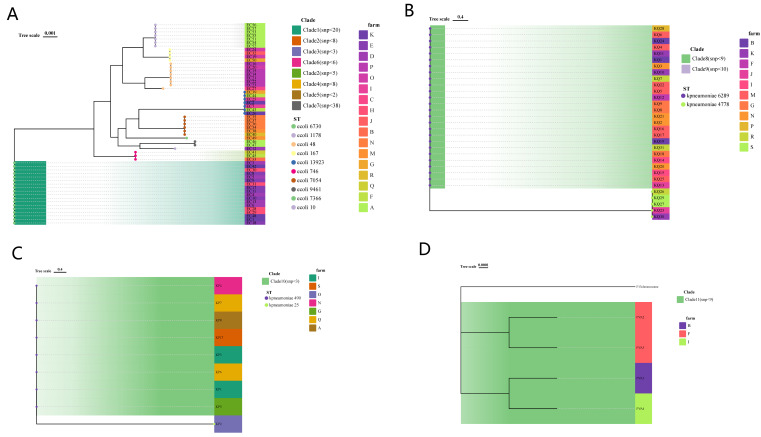
SNP phylogenetic tree of *E. coli* (**A**), *Klebsiella pneumoniae* (**B**), *Klebsiella quasipneumoniae* (**C**), and *Providencia vermicola* (**D**) in this study. MLST, source farms were illustrated.

**Figure 3:**
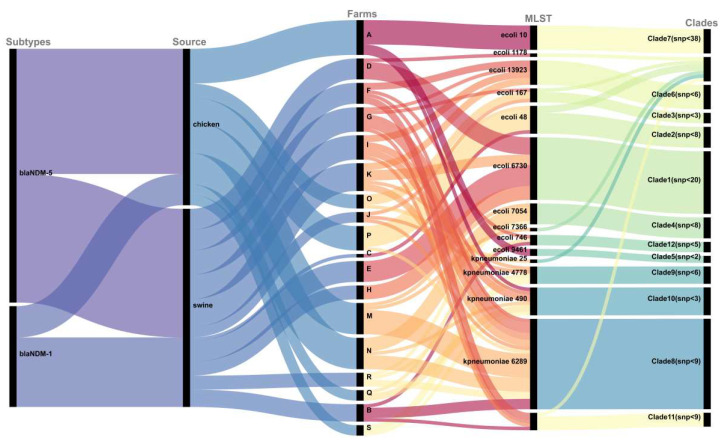
Sankey diagram of *bla*_NDM_ variants of 102 isolates, source farms, ST types, and phylogenetic tree branches. The diameter of the line is proportional to the number of isolates.

By performing MLST on 102 strains, it was found that 57 *E. coli* strains in this study could be classified into 10 different sequence types (STs), among which ST6730 (18/57), ST48 (8/57), ST10 (7/57), ST13923 (7/57), and ST7054 (6/57) were common, accounting for approximately 80.7% of the total. The remaining about one-fifth are ST167, ST7366, ST9461, ST1178, and ST746. *K. pneumoniae* and *K. quasipneumoniae* were assigned to four ST types, namely ST490 (8/9), ST5 (1/9), ST148 (26/31), and ST51 (5/31). *E. coli* ST13923 and *K. pneumoniae* ST51 and ST148 were considered new ST types. The constructed core genome phylogenetic tree was very similar to the MLST results, with the strains sharing the same ST type clustering together, and the number of SNPs was limited. Interestingly, we found that *P. vermicola* from three farms in Sichuan Province and Yunnan Province had a close evolutionary relationship (SNP < 35) with a strain (P13) isolated from the blood of Wenzhou Hospital patients in China (Figure 2 and Figure 3 and Supplementary Tables S1, S2 and S5) [32].

**Figure 4:**
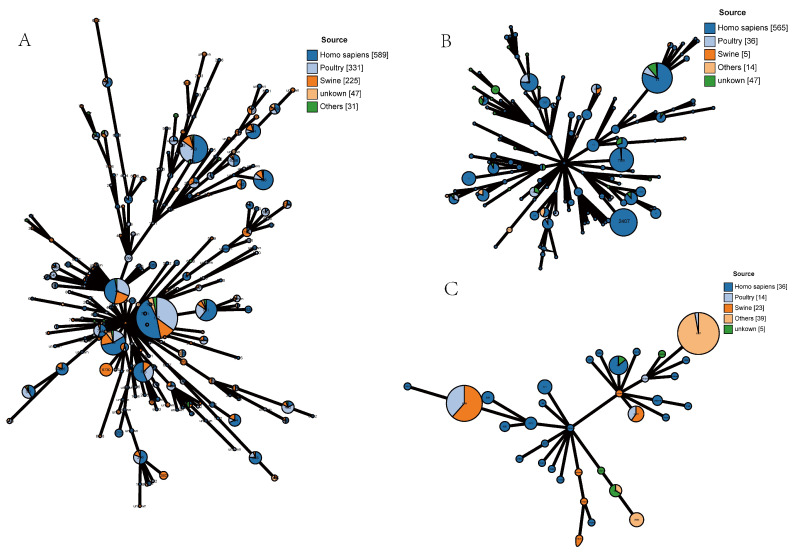
MLST allele profiles of *E. coli*, *Klebsiella pneumoniae,* and *Klebsiella quasipneumoniae* in this study with corresponding *bla*_NDM_-positive strains from China in the NCBI gene assembly database. (**A**) 57 *E. coli* strains with 1166 *bla*_NDM_-positive *E. coli* isolates from the database; (**B**) 9 *Klebsiella pneumoniae* strains with 658 *bla*_NDM_-positive *Klebsiella pneumoniae* isolates from the database; (**C**) 31 *Klebsiella quasipneumoniae* strains and 86 *bla*_NDM_ positive *Klebsiella quasipneumoniae* isolates from the database. Dark blue: human-associated source; light blue: avian-associated source; orange: pig-associated source; yellow: others, green: unknown.

**Figure 5:**
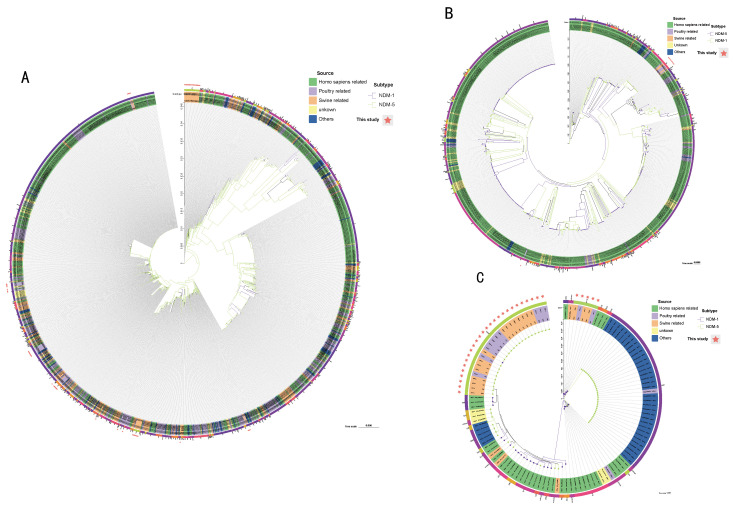
Maximum likelihood phylogenetic tree of *E. coli*, *Klebsiella pneumoniae,* and *Klebsiella quasipneumoniae* in this study with corresponding *bla*_NDM_-positive strains from China in the NCBI gene assembly database. (**A**) 57 *E. coli* strains with 1166 *bla*_NDM_-positive *E. coli* isolates in the database; (**B**) 31 *Klebsiella pneumoniae* strains with 658 *bla*_NDM_-positive *Klebsiella pneumoniae* isolates in the database; (**C**) 9 *Klebsiella quasipneumoniae* strains with 86 *bla*_NDM_-positive *Klebsiella quasipneumoniae* isolates in the database. The outermost circle is the MLST of the strain. Green: human-associated source; light pink: avian-associated source; orange: pig-associated source; yellow: others; blue: unknown.

To further understand the epidemiological characteristics of *bla*_NDM_-positive strains, we analyzed the strains in this study together with *bla*_NDM_-positive strains from China in the NCBI database. Among *E. coli* isolates, ST167, ST10, ST48, ST746, ST617, and ST410 were the most prevalent STs, and they were all found to exist in humans, pigs, chickens, and other places. However, ST6730 was only isolated from pigs (Figure 4, Supplementary Table S2). The phylogenetic tree showed the phylogenetic relationships among strains from different sources, and some animal-derived isolates and human-derived isolates were found to have a very close evolutionary relationship (Figure 5, Supplementary Table S6). For example, there were only two SNP differences between the core genomes of strains EC51-EC57 in this study and strain Ec-03 (GCA_001893865.1) isolated from human urine in 2012. Strains EC19, EC24, EC32, and EC50 and strains E1-E6 (PRJNA298278, isolated in 2014 from the blood of a patient in a hospital in Zhejiang Province), strain SCEC020016 (GCA_002164985.1, isolated in 2016 from a human in a hospital in Sichuan Province), strain E4903 (GCA002265005.1, isolated from human blood of a hospital in Henan Province in 2015), and strain L743 (GCA_002870165.1, isolated from human feces of a hospital in Hangzhou City in 2017) share SNPs less than 24. For *K. pneumoniae* and *K. quasipneumoniae*, the prevailing STs were ST2407, ST789, ST11, ST37, ST6289, ST1697, ST414, and ST4778 (Figure 4). Combined with the corresponding phylogenetic tree (Figure 5), overall, unlike that with *E. coli*, both strains showed a strong host specificity, and the evolutionary relationship between strains from different sources was distant.

### 3.4. Genetic Environments and Transferability of bla_NDM_

**Figure 6:**
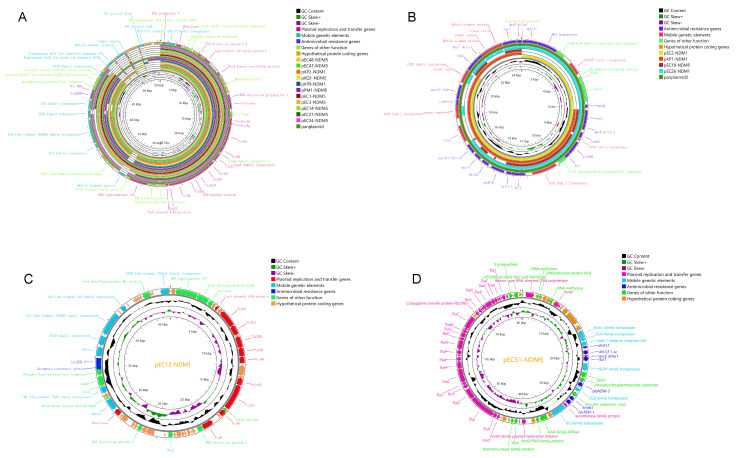
Circle plots and comparative analysis of plasmids carrying *bla*_NDM_. (**A**) Comparative analysis of 11 IncX3 plasmids. (**B**) Comparative circle plots of pEC2-NDM1, pKP1-NDM1, pEC12-NDN5, and pEC26-NDM1 plasmids. (**C**) Circle plots of pEC12-NDM1 plasmid. (**D**) Circle plots of pEC51-NDM5 plasmids. GC content, GC backbone, plasmid replication and transfer-related genes, MGE-related genes, antibiotic resistance genes, other genes and pan-plasmids were illustrated.

**Figure 7:**
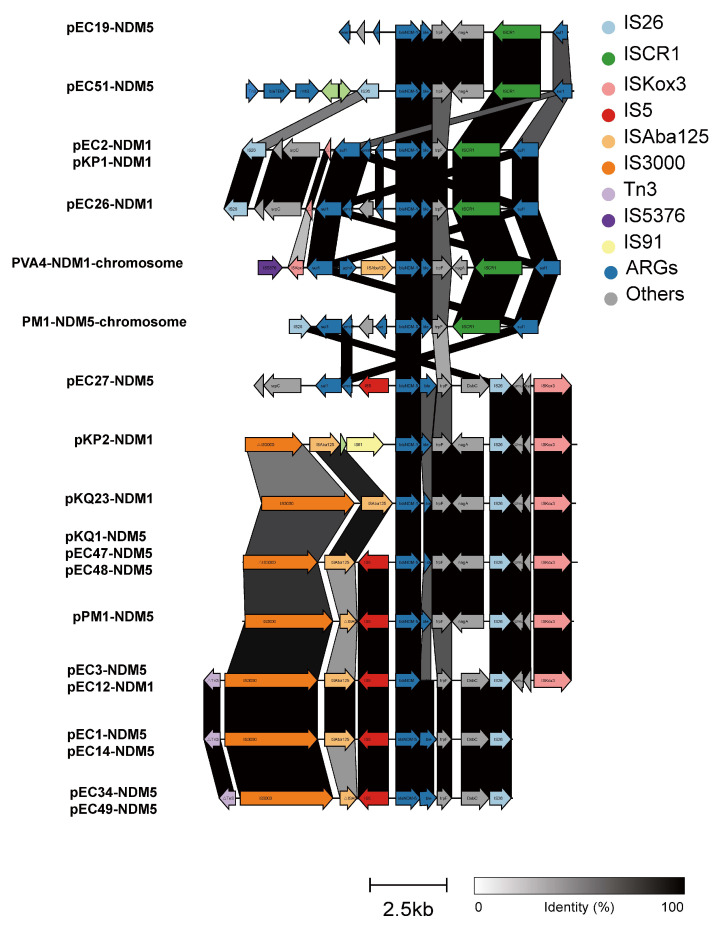
Comparative analysis and annotation of the gene environment of *bla*_NDM_ in length-long sequenced strains. The degree of shading represents the identity of the nucleic acid sequences.

Based on the next-generation sequencing results of 102 strains, we selected the most similar plasmids by comparing the contigs containing the *bla*_NDM_ gene with the NCBI database as a reference. The *bla*_NDM_ genes in genetically distant strains share the same genetic environment. Complementary gaps were sequenced by PCR and nanopore long-read sequencing to obtain the complete genetic environment of the *bla*_NDM_ genes. The *bla*_NDM_ genes are located on the chromosome (Integrative Conjugative elements, ICEs) in *P. vermicola* and exists on both the chromosome (insertion sequence, IS) and the plasmid in *P. mirabilis*. The *bla*_NDM_ genes in the other sequenced strains were found to all be located on the plasmids (Figure 6 and Figure 7). Of the 18 different plasmids, 13 belonged to the IncX3 type, pEC51-NDM-5 (80348 bp) belonged to the IncFII type, and the other four plasmids were not classified. The molecular weights of the complete plasmids range from 20817-80348bp (Supplementary Table S7). The genetic environment could be divided into 14 types, as follows: *bla*_NDM_-(Δ)ble-traF was the most conserved genetic structure, and (Δ)nagA often existed downstream of traF. In general, there were two types of mobile genetic elements downstream of *bla*_NDM_-(Δ)ble-traF, namely (I) ISCRⅠ and (II) IS26-umuD-(ISKox3). The upstream environment was found to be more complex, including IS26-ΔISKox3, IS5376-ΔISKox3-ISABa125, IS3000-ISABa125, ΔIS3000-ISABa125-IS91, IS26, and IS3000-(Δ) IS30-IS5 (Figure 7). We also determined the frequency of the conjugative transfer of *bla*_NDM_ drug-resistance genes in the selected strains and found that the NDM gene from most strains could be transferred to the recipient strain EC600 (Supplementary Table S7), and the conjugative frequency ranged from 1.5 × 10^−8^ to 5.3 × 10^−6^. The non-conjugative plasmid pEC2-NDM-1(20817 bp) without conjugation-associated genes carried by *E. coli* could also be transferred to the recipient strain.

## 4. Discussion

Since its discovery in India in 2009, the *bla*_NDM_ resistance gene has drawn much attention because it confers resistance to the therapeutic antibiotics carbapenems, which are crucial in clinical settings [6]. There have been numerous studies on the prevalence of the *bla*_NDM_ gene in China, but reports on the prevalence of the *bla*_NDM_ resistance gene in pig and chicken farms in the southwest are limited [9,32,33]. In 2021, we isolated and screened *bla*_NDM_-positive isolates from farms in the provinces of Sichuan and Yunnan and analyzed their epidemic characteristics.

The carriage rate of *bla*_NDM_-positive strains in pigs and chickens were 6.05% and 7.89%, respectively. These were higher than those reported in previous studies [12], indicating a high risk of *bla*_NDM_-positive strain transmission to humans via livestock and poultry. A possible reason for this high prevalence is that *bla*_NDM_ can not only confer resistance to carbapenem antibiotics, but also to veterinary cephalosporin antibiotics. Therefore, the use of cephalosporins on farms may provide selection pressure. Moreover, their high occurrence could result from co-selection with other drug-resistance genes. Notably, *bla*_NDM_-positive strains are all multi-drug resistant bacteria that carry multiple antibiotic-resistance genes, and the antibiotic resistance situation is very serious. Given these circumstances, we need to strengthen the monitoring of *bla*_NDM_ drug-resistance genes.

The transmission and spread of *bla*_NDM_ genes are greatly facilitated by *E. coli* and *Klebsiella* spp., which are significant reservoirs of these drug resistance genes, in line with earlier research findings [9,12,34]. The results of antibiotic susceptibility experiments and the prediction of drug resistance genes showed that all bacterial strains were MDR and that different bacterial species had different patterns of antibiotic resistance. In previous studies, *bla*_NDM_-positive strains showed a low susceptibility to tigecycline; however, the high rate of tigecycline resistance in *K. quasipneumoniae* reported in this investigation suggests that this drug might become less effective [9,34]. Polymyxin B is also an effective antibiotic for the treatment of infections caused by *bla*_NDM_-positive strains.

There are many plasmid types in these bacteria, which is helpful for the potential transfer of *bla*_NDM_ or other drug-resistance genes. For example, the *bla*_NDM_ gene could be transferred between different plasmids through the IS. Furthermore, conjugative plasmids or ICEs can help non-conjugative plasmids containing the *bla*_NDM_ gene move between bacteria; for example, the pEC2-NDM-1 plasmid (20817 bp) of strain EC2 in subsequent conjugative transfer experiments could be transferred in this way.

In this study, the same species of bacteria isolated from the same farm showed a limited genetic distance, indicating that vertical transmission is the main mode of transmission of the *bla*_NDM_ gene within this farm. Moreover, the related strains were also common among different farms, indicating the transmission of *bla*_NDM_-positive strains in this region. The *bla*_NDM_-positive *E. coli* in this study had more genetic diversity than other bacteria, which indicates, to a certain extent, that the *bla*_NDM_ gene might be transferred more frequently among this species, and this might also be caused by the larger base number of *E. coli* bacteria in the environment.

The close relationship between *P. vermicola* and human isolates in this study indicates that this strain might have spread widely throughout China [35]. The results of the joint analysis of strains in this study and *bla*_NDM_-positive strains in the database showed some interesting phenomena; for example, some strains of *E. coli* and Klebsiella spp. from human and animal sources were determined to have a high genetic similarity (SNP < 10), indicating that *bla*_NDM_-positive strains are transmitted between farm animals and humans. Moreover, unlike *Klebsiella* spp., more human- and animal-derived *E. coli* strains share the same ST and have a high genetic similarity, which might indicate that *bla*_NDM_-positive *E. coli* is more frequently transmitted between humans and farm animals. Considering these findings, the surveillance of *E. coli* carrying the *bla*_NDM_ gene should be intensified.

Mobile genetic elements are important for the transmission of *bla*_NDM_. Plasmids and ICEs can carry and transfer *bla*_NDM_ genes [33,36,37]. As in previous studies, IncX3 plasmids were found to be the main carriers of the *bla*_NDM_ gene and drivers of its horizontal transmission, but other types of plasmids, ICEs, and ISs are also non-negligible drivers [15,18,19]. It appears from a more specific genetic context that the *bla*_NDM_ gene, in addition to its most conserved structure (*bla*_NDM_-(Δ)ble-traF), is flanked by variable structures with multiple types of ISs on both sides. The results of this study suggest that ISs, such as IS26, ISCR1, and ISAba125, play important roles in the transfer and recombination of *bla*_NDM_ between different genetic environments. Both ICEs and most plasmids in the experiments could be transferred between bacteria via conjugation, indicating that these *bla*_NDM_ genes have a strong capacity for horizontal transmission. Although the horizontal transfer of *bla*_NDM_ occurs readily under laboratory conditions, the reasons for not monitoring abundant and diverse *bla*_NDM_ carriers within the same farm are worthy of further exploration.

## 5. Conclusions

Overall, this study provides an in-depth analysis of *bla*_NDM_-positive strains in pig and chicken farms in Southwest China. Carriage rates of *bla*_NDM_ in farm animals on both pig and chicken farms are at a high level. *E. coli* and *Klebsiella* spp. were the main *bla*_NDM_ carriers in the *Enterobacterales*, *bla*_NDM-5_ was the main subtype, and all strains were MDR. Moreover, the IncX3 plasmid was the main carrier of the *bla*_NDM_ gene, and most of it was found to be transferred via conjugation. Phylogenetic analysis suggests that vertical transmission of host strains with limited genetic diversity is the main cause of the spread of *bla*_NDM_ in Sichuan pig and chicken farm animals. The potential threat of reciprocal transmission between *bla*_NDM_-positive isolates of animal and human origins is worrisome. Therefore, further monitoring is urgently needed to explore this transmission and to disrupt the transmission network.

## Data Availability

Not applicable.

## Supplementary Materials

The following supporting information can be downloaded at: https://www.mdpi.com/article/10.3390/microorganisms11092304/s1. Table S1: Information for 102 *Enterobacterales* strains from *bla*_NDM_-positive strains in this study. Table S2: Details of 1911 *Enterobacterales* strains containing *bla*_NDM_-1 or *bla*_NDM_-_1_ resistance genes from China (database: NCBI, Dates to December 2022). Table S3: Resistance profiles of 102 *bla*_NDM_-positive *Enterobacterales* strains to 16 antibiotics in this study. Table S4: esistance genes and plasmid replicons profiles of 102 *bla*_NDM_-positive *Enterobacterales* strains in this study. Table S5: SNP matrix for 4 classes of strains in this study. Table S6: SNP matrix between strains in this study and homozygous strains from the database. Table S7: The frequency of conjugative transfer of *bla*_NDM_ gene in the selected 19 strains.
